# Nano-structured CuO-Cu_2_O Complex Thin Film for Application in CH_3_NH_3_PbI_3_ Perovskite Solar Cells

**DOI:** 10.1186/s11671-016-1621-4

**Published:** 2016-09-15

**Authors:** Lung-Chieh Chen, Cheng-Chiang Chen, Kai-Chieh Liang, Sheng Hsiung Chang, Zhong-Liang Tseng, Shih-Chieh Yeh, Chin-Ti Chen, Wen-Ti Wu, Chun-Guey Wu

**Affiliations:** 1 Department of Electro-Optical Engineering, National Taipei University of Technology, 1, Section 3, Chung-Hsiao E. Road, Taipei, 106 Taiwan; 2 Research Center for New Generation Photovoltaics, National Central University, Taoyuan, 32001 Taiwan; 3 Department of Optics and Photonics, National Central University, Taoyuan, 32001 Taiwan; 4 Institute of Chemistry, Academia Sinica, Taipei, 11529 Taiwan

**Keywords:** Nano-structured, CuO-Cu_2_O complex-based, CH_3_NH_3_PbI_3_ perovskite

## Abstract

Nano-structured CuO-Cu_2_O complex thin film-based perovskite solar cells were fabricated on an indium tin oxide (ITO)-coated glass and studied. Copper (Cu) thin films with a purity of 99.995 % were deposited on an ITO-coated glass by magnetron reactive sputtering. To optimize the properties of the nano-structured CuO-Cu_2_O complex thin films, the deposited Cu thin films were thermally oxidized at various temperatures from 300 to 400 °C. A CH_3_NH_3_PbI_3_ perovskite absorber was fabricated on top of CuO-Cu_2_O complex thin film by a one-step spin-coating process with a toluene washing treatment. Following optimization, the maximum power conversion efficiency (PCE) exceeded 8.1 %. Therefore, the low-cost, solution-processed, stable nano-structured CuO-Cu_2_O complex thin film can be used as an alternative hole transport layer (HTL) in industrially produced perovskite solar cells.

## Background

Organic–inorganic hybrid perovskite (such as CH_3_NH_3_PbX_3_, X = I, Cl, Br) solar cells has attracted much attention because of its superior photovoltaic performance, including excellent light-harvesting ability and potential applications [1–6]. In previous reports, the high power conversion efficiency (PCE) of perovskite solar cells was achieved when a conducting polymer PEDOT:PSS thin film that was formed using TiO_2_ nano-particles that had been sintered at high temperature was used as the electron transport layer (ETL) (hole transport layer (HTL)) [7–9]. However, a trade-off must be between fabrication cost and device stability, impeding the development of commercialized solar cells. Inorganic p-type materials as hole transport media have the double advantage of excellent chemical stability and simplicity of preparation [10–12]. Along with some inorganic HTLs (copper iodide (CuI) [13], copper thiocyanate (CuSCN) [14–16], graphene oxide (GO) [17], nickel oxide (NiO) [18], cuprous oxide (Cu_2_O), and copper oxide (CuO) [19] have attracted substantial interest owing to their direct gaps of 1.9–2.2 eV[20–22], and these have been widely used as HTLs in solar cells. Cu_2_O and CuO thin films can be prepared by various methods, including reactive sputtering [23], electrochemical deposition [24–27], chemical dissolution [28–30], thermal oxidation [31], and successive ionic layer adsorption and reaction (SILAR) method [12, 32].

In this work, CuO-Cu_2_O complex thin films were prepared from thermally oxidized Cu thin films to form HTLs for use in perovskite solar cells. The structural, optical, and surface properties of CuO-Cu_2_O complex thin films were investigated to elucidate the performance of CuO-Cu_2_O complex thin film-based photovoltaics. The device with an optimized CuO-Cu_2_O complex thin film as the HTL exhibited superior photovoltaic performance, revealing that the CuO-Cu_2_O complex thin film is a potential inorganic HTL for use in perovskite solar cells.

## Methods

**Fig. 1:**
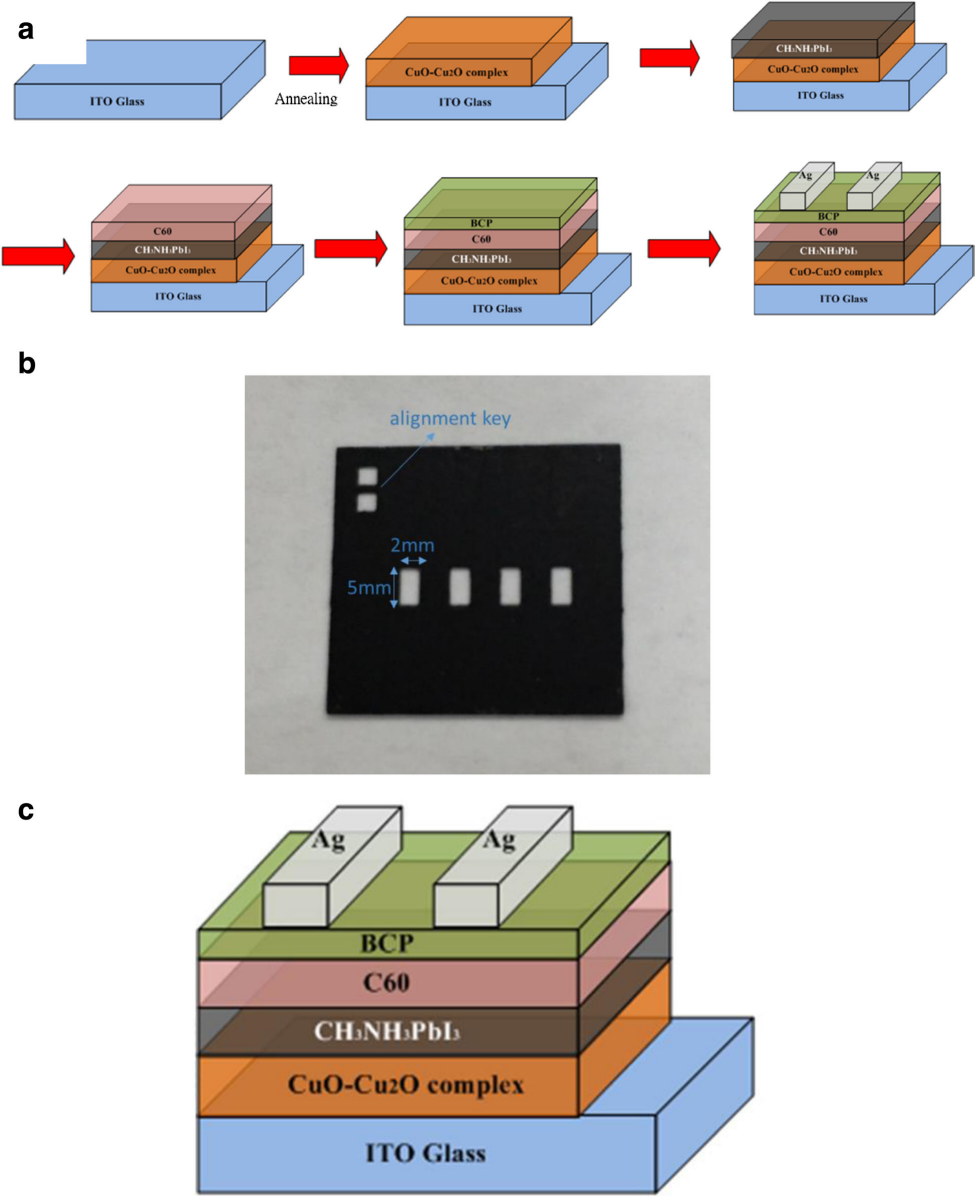
**a** Preparation of nano-structured CuO-Cu_2_O complex films, **b** photograph image of shadow mask, and **c** structure of device

Firstly, Cu layers were deposited on an indium tin oxide (ITO) glass substrate by RF magnetron reactive sputtering from Cu targets in gaseous argon (Ar) at a flow rate of 15 sccm and a stable working pressure of 3 × 10^−3^ Torr. The nano-structured CuO-Cu_2_O complex thin films were formed by thermally oxidizing Cu on ITO substrate at various annealing temperatures from 300 to 400 °C for 1 h in air, and these acted as HTLs. 1.25 M Pbl_2_ and 1.25 M methylammonium iodide (MAI) were dissolved in a cosolvent mixture of dimethyl sulfoxide (DMSO) and γ-butyrolactone (GBL) (vol. ratio = 1:1), and the resulting solution was used as the perovskite precursor solution. The CH_3_NH_3_PbI_3_ perovskite precursor was spin-coated on top of the nano-structured CuO-Cu_2_O complex/ITO/glass and underwent in situ non-polar solvent washing treatment [1–3] in a glove box that was filled with highly pure nitrogen. The CH_3_NH_3_PbI_3_ perovskite precursors were then coated onto the CuO-Cu_2_O complex/ITO/glass samples in two consecutive spin-coating steps at 1000 and 5000 rpm for 10 and 20 s, respectively. At 5000 rpm for 20 s, the wet spinning film was quenched by dropping 100 μl of anhydrous toluene onto it. After spin coating, the film was annealed at 100 °C for 5 min. Finally, C_60_, BCP, and the Ag electrode were sequentially deposited by thermal evaporation at a base pressure of 5 × 10^−6^ Pa. The thicknesses of C_60_, BCP, and Ag were 50, 5, and 100 nm, respectively. Figure 1a presents the steps in the preparation of CuO-Cu_2_O complex thin film-based perovskite solar cells. The C_60_ thin film, CH_3_NH_3_PbI_3_ perovskite thin film, and CuO-Cu_2_O complex thin film in the cell structure acted as the ETL, the active layer, and the HTL, respectively. A shadow mask firmly covered the sample to define an active area of 0.5 cm × 0.2 cm during C_60_/BCP/Ag deposition. Figure 1b shows the photo image of the shadow mask. Figure 1c schematically depicts the complete structure.

The crystal characteristics of the CuO-Cu_2_O complex thin films were determined using an X-ray diffractometer (XRD) (D8, Bruker). Raman scattering spectroscopy was used to analyze the CuO-Cu_2_O complex thin films. A field-emission scanning electron microscope (FESEM) (LEO 1530) was used to observe the cross-section and surface morphology of the cells. The current density–voltage (J-V) characteristics were measured using a Keithley 2400 programmable source/meter unit under irradiation by a 1000 W xenon lamp. To evaluate the photovoltaic performance, the irradiation power density on the surface of the sample was calibrated as set to 1000 W/m^2^. Photoelectron emission measurement (manufactured by Riken Keiki, Model AC-2) was performed in the air and at room temperature.

## Results and Discussion

Table 1 presents the Hall measurements of the CuO-Cu_2_O thin films. The increase in the carrier mobility at 250–300 °C could be attributed to an improvement in the crystallinity as well as a decrease in the carrier density because carrier mobility is generally affected by grain boundary scattering and by impurity scattering due to the native defects. However, as the annealing temperature increased to 400 °C, the mobility in the sample decreased from 33.5 to 17.9 cm^2^/Vs for owing to the decrease in the size of the grains caused by the phase transformation from Cu_2_O to CuO. As the thermal annealing temperature increased, the reduction of resistivity of the CuO-Cu_2_O complex films post-annealing may be attributed to both the carrier concentration and carrier mobility were decreased gradually, resulting in an increase in resistivity. The reduction of mobility is attributable to the transportation of carriers from one grain to another grain.

**Table 1:** Hall measurements of CuO-Cu_2_O complex films

Annealing temp. (°C)	Resistivity (Ω-cm)	Carrier concentration ×10^17^ (cm^−3^)	Mobility (cm^2^/V-s)
As-deposited	2.1	2.9	10.3
300	1.5	2.1	19.3
350	2.0	9.2	33.5
400	9.7	3.6	17.9

**Fig. 2:**
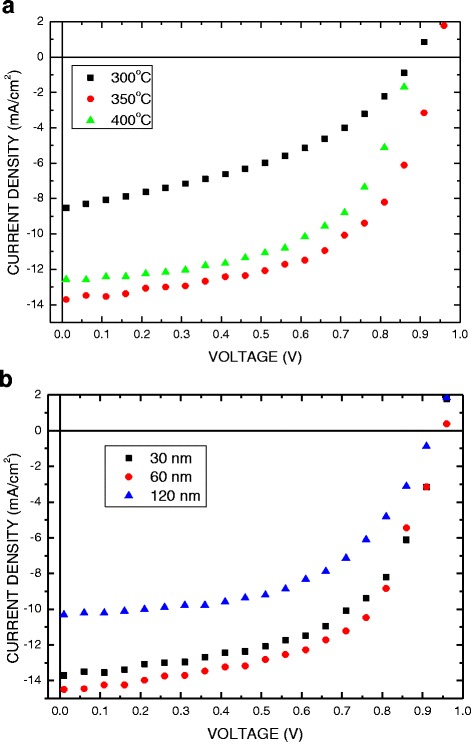
Current–voltage (J-V) characteristics of perovskite solar cell that was constructed using CuO-Cu_2_O complex thin film under simulated illumination with a light intensity of 100 mW/cm^2^ (AM 1.5). **a** Thermal oxidation temperatures. **b** The thicknesses of CuO-Cu_2_O complex thin film

Figure 2 plots the J-V curves for the CuO-Cu_2_O complex thin film-based perovskite solar cells under 100 mW/cm^2^ illumination (AM 1.5G). Tables 2 and 3 list the characteristic parameters of these devices. The PCE is improved from 3.15 to 7.32 % as the thermal oxidation temperature increases from 300 to 350 °C, mainly owing to the increase in the photo-generated carriers extracted and injected into the electrode caused by the carrier mobility and series resistance in the device, and then to result in the increase in the short-circuit current density (J_SC_). The PCE decreases from 7.32 to 6.43 % as the thermal oxidation temperature increased from 350 to 400 °C. As listed in Table 2, for the device annealing at 400 °C, the series resistance (Rs) increases and the shunt resistance (Rsh) decreases. The degradation of the performance may be attributed to the degradation of ITO electrode and leakage between CuO-Cu_2_O complex and perovskite layer. The PCE of CuO-Cu_2_O complex thin film-based perovskite solar cells is improved from 7.32 to 8.10 % as the thickness of CuO-Cu_2_O complex thin film increased from 30 to 60 nm. However, further increasing the thickness of the CuO-Cu_2_O complex thin film to 120 nm reduced the PCE from 8.10 to 5.20 % due to the series resistance effect in the cell, as listed in Table 3. According to the J-V curve and PCE value, the optimized thickness of the CuO-Cu_2_O complex thin film is 60 nm.

**Table 2:** Parameters of CuO-Cu_2_O complex thin film-based perovskite solar cells following thermal oxidation of the film at various temperatures

Annealing temp. (°C)	V_OC_(V)	J_SC_(mA/cm^2^)	FF(%)	Eff(%)	Rs(Ω)	R_sh_(Ω)
300	0.88	8.56	41. 42	3.15	25.8	234
350	0.95	13.88	55.54	7.32	7.9	738
400	0.89	12.71	56.89	6.43	12.5	698

**Table 3:** Parameters of CuO-Cu_2_O complex thin film-based perovskite solar cells with films of different thicknesses

CuO-Cu_2_O complex thickness (nm)	V_OC_(V)	J_SC_(mA/cm^2^)	FF(%)	Eff(%)	Rs(Ω)	R_sh_(Ω)
30	0.95	13.88	55.54	7.32	12.9	410
60	0.96	14.40	58.61	8.10	10.3	789
120	0.93	10.28	54.45	5.20	21.9	338

**Fig. 3:**
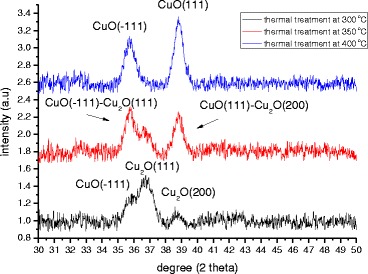
XRD diffractograms of CuO-Cu_2_O complex thin film following thermal oxidation at various temperatures

**Fig. 4:**
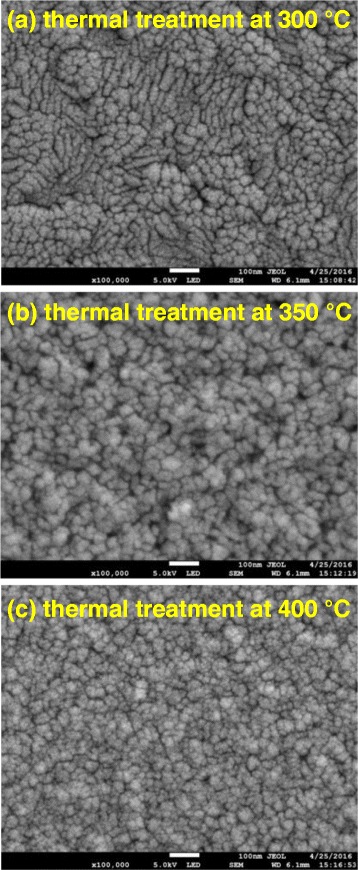
FESEM images of CuO-Cu_2_O complex thin films fabricated using thermal oxidation at various temperatures. **a** Thermal treatment at 300 °C. **b** Thermal treatment at 350 °C. **c** Thermal treatment at 400 °C

**Fig. 5:**
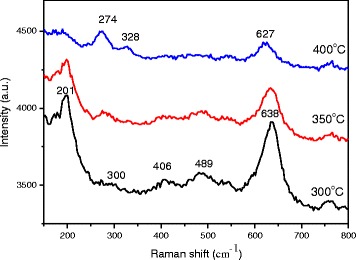
Raman scattering spectra of CuO-Cu_2_O complex thin films under 473-nm excitation

**Fig. 6:**
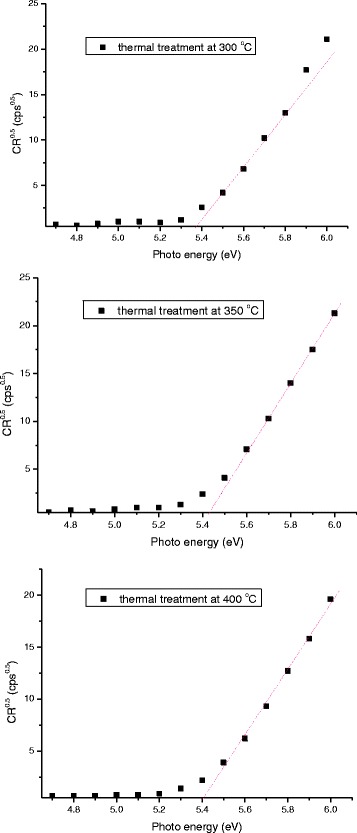
Photoelectron emission spectra of CuO-Cu_2_O complex thin films

In this study, the X-ray diffraction technique was used to elucidate the crystal characteristics of CuO-Cu_2_O complex thin films. To determine the relationship between thermal oxidation temperature and mechanism, the phase of the CuO-Cu_2_O complex thin films was identified. Figure 3 shows the XRD patterns of CuO-Cu_2_O complex thin films that were deposited on a glass substrate. XRD experimental results demonstrate that the crystalline phases of CuO and Cu_2_O were formed at different thermal oxidation temperatures. Diffraction peaks at 35.84° and 38.85° corresponded to the (−111) and (100) planes of the cubic-structured CuO, and diffraction peaks at 36.8° and 38.63° corresponded to the (111) and (200) planes of the cubic-structured Cu_2_O. A broad diffraction peak at 35.84° was obtained from the Cu/ITO/glass sample following thermal annealing at 350 °C. This peak may be attributed to a complex layer that comprised the (−111) plane of CuO and the (111) plane of Cu_2_O. The XRD patterns of Cu_2_O (CuO) gradually decrease (increase) as the thermal annealing temperature increased from 300 to 400 °C, indicating that the crystalline Cu_2_O is completely converted to crystalline CuO. The crystal domain size *G* was calculated according to the Scherrer’s equation: [33]

1 $$ G=\frac{0.9\lambda }{\beta \cos \theta } $$

where *G*, *λ*, *β*, and *θ* denote the grain size, the X-ray wavelength, the full width at half maximum (FWHM) in radians, and the Bragg angle of CuO or Cu_2_O peak, respectively. The phase transformation from crystalline Cu_2_O to crystalline CuO reduces the crystal domain size from 31.83 to 29.39 nm, and thereby increases the carrier mobility of the CuO-Cu_2_O complex thin films, improving the carrier collection at the CH_3_NH_3_PbI_3_/CuO-CuO_2_ complex interface. However, the phase transformation reduces the grain size and surface roughness of the CuO-Cu_2_O complex thin films, as presented in Fig. 4, which presents FESEM images of the Cu layers that had undergone thermal oxidation at various annealing temperatures. More flat surface favors the coverage of the subsequently coated perovskite layer and C_60_ layer on the Cu-CuO_2_ complex thin films to form the continuous films and so improves the fill factor of the perovskite solar cells (Table 2). The image of the Cu layer that underwent thermal treatment at 350 °C reveals that the surface of the film consisted of small particles. The mean particle size was approximately 20 nm, as displayed in Fig. 4a. When the annealing temperature exceeded 300 °C, thin oxides were formed, particularly in the grain boundary regions, implying that they were formed by fast diffusion processes, as presented in Fig. 4. To elucidate the relation between the crystal domain size and the grain size in CuO-Cu_2_O complex thin films, the Raman scattering spectra of CuO-Cu_2_O complex thin films were measured, as shown in Fig. 5. According to two relevant investigations [34, 35], the five peaks at 201, 300, 406, 489, and 638 cm^−1^ and the three peaks at 274, 328, and 627 cm^−1^ are the Raman fingerprints of Cu_2_O and CuO, respectively. The Cu_2_O phase is completely converted to the CuO phase as the annealing temperature increased from 300 to 400 °C, revealing that the Cu_2_O is converted to CuO. The results are consistent with the results of XRD patterns in Fig. 3. Therefore, the grain size (Fig. 4) is reduced when the annealing temperature increased from 350 to 400 °C owing to the phase transformation. Figure 6 presents the photoemission spectra of Cu-Cu_2_O complex thin films, which were used to determine their work functions. The work function of a CuO-Cu_2_O complex thin film is proportional to the Voc of the perovskite solar cell in which it is used.

## Conclusions

This study examined the characteristics of a nano-structured CuO-Cu_2_O complex thin-film for use in perovskite solar cells. A CH_3_NH_3_PbI_3_ perovskite absorber was fabricated on top of a CuO-Cu_2_O complex thin film in a one-step spin-coating process, which was followed by toluene washing treatment. The work function of the Cu-Cu_2_O complex thin film varied with the annealing temperature. The work function of a CuO-Cu_2_O complex thin film is proportional to the Voc of the perovskite solar cell in which it is used. Following optimization, the maximum power conversion efficiency (PCE) exceeded 8.1 %. Therefore, the low-cost, solution-processed, stable nano-structured CuO-Cu_2_O complex thin film can be used in alternative hole transport layers (HTLs) in industrially produced perovskite solar cells.

## Abbreviations

Cu_2_O: cuprous oxide
CuO: copper oxide
ETL: electron-transporting layer
FESEM: field-emission scanning electron microscope
HTL: hole transport layer
ITO: indium tin oxide
J-V: current density–voltage
PCE: power conversion efficiency
XRD: X-ray diffractometer
